# Monte Carlo simulation for the prediction of precision of absorbance measurements with a miniature CCD spectrometer

**DOI:** 10.1155/S1463924603000063

**Published:** 2003

**Authors:** E. P. Tsaousoglou, S. D. Bolis, C. E. Efstathiou

**Affiliations:** Laboratory of Analytical Chemistry Department of Chemistry University of Athens University Campus Athens GR-157 71 Greece

## Abstract

The precision characteristics of the absorbance measurements obtained with a low-cost miniature spectrometer incorporating an array detector were evaluated. Uncertainties in absorbance measurements were due to a combination of non-uniform light intensity and detector response over the wavelength range examined (350-850 nm), in conjunction with the digitization of the intensity indications and the intrinsic noise of the detecting elements. The precision characteristics are presented as contour plots displaying the expected RSD% of absorbances on the absorbance versus wavelength plane. The minimum RSD% for the spectrometer configuration tested was observed within the 0.2-1.5 absorbance units and 500-750 nm wavelength range. Without invoking signal enhancement features of the data-acquisition program (scan average, higher integration times, smoothing based on averaging the signal detected by adjacent pixels), the attainable precision within this range was 0.4-0.8%. A computer program based on Monte Carlo simulations was developed for the prediction of absorbance precision characteristics under various conditions of measurements.


Journal of Automated Methods & Management in Chemistry
Vol. 25, No. 2 (March–April 2003) pp. 35–42

Taylor & Francis

Taylor & Francis Group

### Monte Carlo simulation for the prediction of precision of absorbance measurements with a miniature CCD spectrometer

**E. P. Tsaousoglou, S. D. Bolis and C. E. Efstathiou***

*Laboratory of Analytical Chemistry, Department of Chemistry, University of Athens, University Campus, Athens GR-157 71, Greece*

*The precision characteristics of the absorbance measurements obtained with a low-cost miniature spectrometer incorporating an array detector were evaluated. Uncertainties in absorbance measurements were due to a combination of non-uniform light intensity and detector response over the wavelength range examined (350–850 nm), in conjunction with the digitization of the intensity indications and the intrinsic noise of the detecting elements. The precision characteristics are presented as contour plots displaying the expected RSD% of absorbances on the absorbance versus wavelength plane. The minimum RSD% for the spectrometer configuration tested was observed within the 0.2–1.5 absorbance units and 500–750 nm wavelength range. Without invoking signal enhancement features of the data-acquisition program (scan average, higher integration times, smoothing based on averaging the signal detected by adjacent pixels), the attainable precision within this range was 0.4–0.8%. A computer program based on Monte Carlo simulations was developed for the prediction of absorbance precision characteristics under various conditions of measurements.*

### Introduction

A number of miniaturized ultraviolet (UV)/visible spectrometers incorporating inexpensive charge coupled devices (CCD) as detecting elements are available from many commercial sources at relatively low cost. Almost all these instruments use optical fibres as guides of the optical signal, and they are usually accompanied by a wide collection of accessories such as a variety of light sources, cuvette holders, flow cells and dipping-type photometric probes, expanding considerably the spectrum of their potent applications. Owing to their compact size, rigid structure, the absence of moving parts and ability to provide practically simultaneous optical measurements over a wide range of wavelengths, these devices are particularly suitable for automated analytical systems. Typical applications include flow injection systems based on spectrophotometric detection [1, 2] and chemiluminescence measurements [3, 4]. A universal microflow analyser based on sequential and bead injection 'lab-on-valve' using a CCD spectrometer for absorbance and fluorescence measurements has also been described [5]. Still, their ability to provide precise optical measurements must be thoroughly examined before their incorporation into fully automated stand-alone systems. Worsfold and his group have performed a thorough assessment of key instrumental parameters (wavelength repeatability, photometric linearity, photometric precision and instrumental drift) of a typical miniature CCD spectrometer (Ocean Optics PSD-1000) in the prospect of using this device for remote and long-term deployment [6].

The present paper presents the results of a thorough investigation on the short-term photometric precision attainable with a similar miniature spectrometer. A simulation program was developed for the prediction of the expected precision of absorbance measurements over the wavelength range 350–850 nm and the absorbance range 0–2.5. This assessment was deemed as an essential step before incorporating this device as a detector to a Sequential-Injection Analyzer and the design of control and data-acquisition software for performing multicomponent analysis and chemometric studies. A prior knowledge of the intrinsic or 'best case' uncertainties due to the detecting system limitations allows the investigator to locate and fix other sources of random errors such as those due to chemistry and/or other parts of the overall instrumentation.

### Experimental

### *Reagents*

Stock solutions (0.50 M) of copper(II), cobalt(II), nickel(II), chromium(III) sulfate or nitrate salts were prepared by dissolving the appropriate amounts of the solid hydrated salts in 0.50 M sulfuric acid. The stock solutions were diluted as necessary with 0.10 M sulfuric acid to obtain solutions covering a wide range of absorbances (0.0–2.5) over the wavelength range (350–850 nm). Solutions (0.10 M) of praseodymium(III) and erbium(III) chlorides in 0.10 M hydrochloric acid were used to check the accuracy of the wavelength scale.

### *Instrumentation*

The miniature spectrometer tested was a single-channel fibreoptic spectrometer (AVS-S2000, Avantes, Eerbeek, The Netherlands). It was based on the Sony ILX511 reduction type highly sensitive 2048-pixel CCD linear image sensor clocked at 2 MHz. The spectrometer input channel was fitted with a collimating lens (L2) and a 50 μm slit (SLIT-50). The spectrometer uses a diffraction grating in an asymmetric crossed Czerny–Turner configuration. The analogue signal created by each light-sensing pixel of the CCD was digitized by a 12-bit A/D

* To whom correspondence should be addressed. e-mail cefstath@chem.uoa.gr

*Journal of Automated Methods & Management in Chemistry* ISSN 1463–9246 print/ISSN 1464–5068 online © 2003 Taylor & Francis Ltd
http://www.tandf.co.uk/journals
DOI: 10.1080/1463924031000095652

converter (1 MHz sampling frequency) located on a card (ADC1000) plugged in a free ISA-bus slot of a PC IBM-compatible computer (μP: 80486).

The light source (HL-2000) was fitted with a long life tungsten-halogen 7 W (5 VDC/1.4 A) bulb, with light intensity stable within ±0.5%, and spectral range of 360–1100 nm (bulb colour temperature 2960 K). The cell used was a Micro Flow Z-type cell with an optical path of 10 mm and volume 18 μl. The cell was connected with the light source and the spectrometer input with two fibreoptic cables FC-UV400-1-FIA-SR (i.d. 400 μm, wavelength range 200–800 nm) fitted with SMA905 connectors.

Touching the fibreoptic cables close to the flow cell caused abrupt changes in the transmitted light intensity measurements due to misalignment of the optical beam. Therefore, it was necessary to align and fasten tightly all optical connections close to the flow cell on a rugged table constructed with $\frac{1}{2}$-inch thick Plexiglas plates. The flow cell was filled with the absorbing or reference aqueous solutions by slow manual injections using a 25-ml syringe. Suction of the solutions through the cell was avoided to prevent the formation of air bubbles within the optical path.

### *Software*

The Avantes SpectraWin version 5.0 Full was installed and used as the data-acquisition program. The Excel 2000 (Microsoft Corporation, Redmont, WA, USA) and Origin 6.0 (Microcal Software, Inc., now OriginLab, Northampton, MA, USA) were used for data treatment and non-linear regression analysis. A home-built program written in Delphi 5.0 (Borland Inprise, Scotts Valley, CA, USA) was used for the simulation studies and drawing of the RSD% of absorbance measurements versus absorbance and wavelength contour plots.

### *Procedures*

*Set-up of the data collection parameters: adjustments with the reference solution.* Unless otherwise stated, all measurements (dark, reference and sample spectra) were obtained without invoking any of the signal-to-noise enhancement features provided by the data-acquisition software.

Even with the minimum integration time some overexposure occurred around the wavelength range of maximum relative sensitivity (600–640 nm) and some attenuation of the incoming light intensity was necessary. The recommended method to alleviate this problem was the use of fibreoptic cables with smaller diameter and/or the use of a neutral density filter. We found it more practical to insert a small glass wool plug in the light-path of the SMA905 connector at the light source with the fibreoptic cable. This plug acts as a thermally and optically stable light absorbing and dispersing element. By trimming the size of this plug, it was easy to adjust the maximum count number (occurred at 621 nm) in the range 3800–3900 with the flow cell filled with water.

*Measurements of RSD% of mean absorbances.* After a warm up of 10 min, the cell was filled with water and the reference spectrum was obtained and stored in the memory. With a shutter blocking the light beam at the light source, the dark spectrum was similarly obtained and stored. The cell was then filled with metal ion solution of various concentrations and the spectrum was recorded. Absorbance measurements were obtained as close as possible to plateau regions to minimize the effects of wavelength instability of the spectrometer on the calculated precision values. The use of organic absorbing compounds was also avoided to eliminate the chance of photodecomposition, since the samples were directly exposed to the bright radiation of the light source and sometimes for a few minutes.

The evaluation of the RSD% of the absorbance indications ($RSD\%_A$) at various wavelengths took place by using the 'History' function of the data-acquisition program. For each wavelength and mean absorbance, a data set of about 1000 consecutive absorbances was collected. The graph of this data set was inspected visually on-screen for drift and/or abrupt changes of absorbances. In either case, the data set was rejected, the source causing the problem located and fixed, and the measurement repeated after obtaining again the dark and reference spectra. The data set was then exported to Excel for the calculation of the corresponding $RSD\%_A$. More than 900 $RSD\%_A$ were calculated over the wavelength range 350–850 nm and the absorbance range 0.0–2.5 by using the metal solutions.

### Results and discussion

### *Factors affecting the precision of absorbance measurements*

Light incident on each pixel of the CCD detector releases electrons, which drift toward a depletion region of the semiconducting material. The accumulated charge is proportional to the light intensity and period (integration time) allowed before the reading cycle. Thus, the depletion region behaves like a capacitor and a voltage appears across it. Upon reading, the voltages for all 2048 pixels are simultaneously transferred to an analogue shift register and they are then sequentially sent to the output pin of the CCD detector. Each voltage indication is digitized to 12 bits, hence the maximum 'count number' expected is $2^{12} - 1 = 4095$, that corresponds to the higher light intensity. A further increase of the light intensity and/or integration time will overexpose the pixels and corresponding count numbers will be clipped at 4095 and they will be useless for the calculation of the absorbance. However, to maintain a signal resolution as high as possible, the maximum indication read with the reference solution must be as close as possible to this figure. The manufacturer recommends an adjustment of the integration time, such that the maximum count over the wavelength range is around 3500 [7].

For all measurements (dark, reference and sample spectra), the minimum integration time (3 ms) was used without any averaging. Smoothing based on averaging of counts measured of $2sm + 1$ adjacent pixels that are practically accepting the same intensity of light was also suppressed (*sm* was set to zero).

The light-sensing pixels of the CCD detector are thermally sensitive; therefore, there is always an accumulation of charge, even under dark. The spectrometer provides an option for a 'dynamic dark correction' by subtracting from all counts the average of dark counts of the first 16 pixels of the CCD which are covered and not exposed to light ('dead pixels'). This option was also not activated. Under these conditions (no counts averaging, no smoothing, no dynamic dark correction), only integer counts are expected in the so-called 'scope mode' (direct readout of the ADC converter output versus the corresponding wavelength).

The spectrometer is a single-channel device; therefore, the 'corrected' transmittance or absorbance spectra of an absorbing solution can only be computed after obtaining the count spectrum of the reference solution ('reference data'), the count spectrum with the optical beam blocked ('dark data') and the count spectrum of the sample solution ('sample data'). All data were stored in the computer memory and the transmittance ($T$) or absorbance ($A$) at a particular wavelength $\lambda$ were calculated:

$$A_\lambda = -\log\ T_\lambda = -\log\left(\frac{C_{\mathrm{sample},\lambda} - C_{\mathrm{dark},\lambda}}{C_{\mathrm{ref},\lambda} - C_{\mathrm{dark},\lambda}}\right). \quad (1)$$

$C_{\mathrm{sample},\lambda}$ and $C_{\mathrm{ref},\lambda}$ are the stored counts, obtained from the charge of the pixel more closely corresponding to wavelength $\lambda$. Both must be corrected by subtracting the corresponding dark counts $C_{\mathrm{dark},\lambda}$.

Under the aforementioned measurement conditions, the resulting spectra may appear highly contaminated with noise. As a typical example in figure 1 are shown the spectra of $Cr^{3+}$ obtained at three different concentration levels. Careful examination reveals that the superimposed noise depends on the wavelength and absorbance range. It should be stressed, however, that less noisy spectra can be obtained by a point-to-point averaging of $N$ spectra. Theoretically, an enhancement of the signal-to-noise proportional to $N^{1/2}$ is expected. A similar enhancement

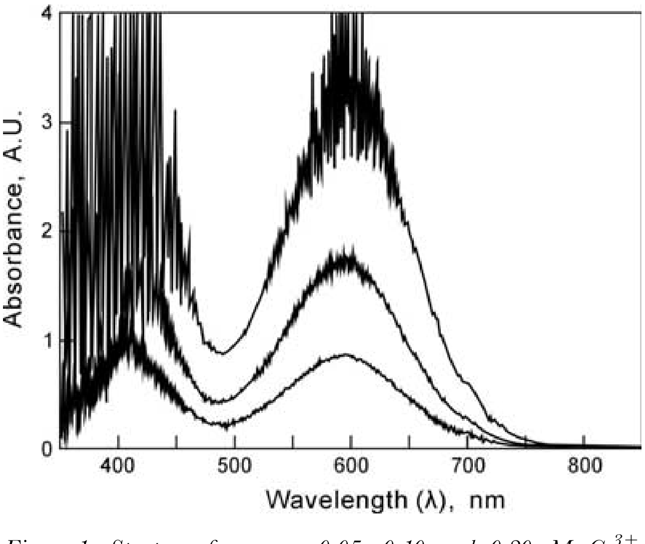

*Figure 1. Spectra of aqueous 0.05, 0.10 and 0.20 M $Cr^{3+}$ solutions.*

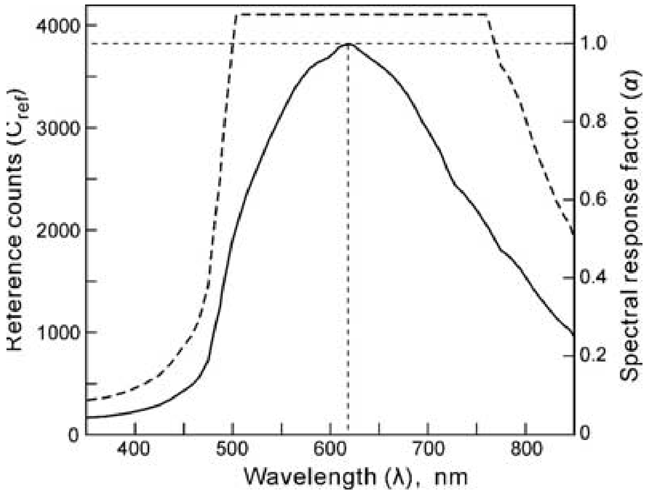

*Figure 2. Typical plot of the number of reference counts versus wavelength (cell filled with pure water) obtained with integration times set to 3 (solid line) and 6 ms (dashed line). In the latter case, the pixels corresponding to the spectral range 500–750 nm were saturated.*

is expected on increasing the integration time, provided that no CCD pixels will be overexposed while the 'sample data' or 'reference data' are obtained. In addition, a drastic S/N enhancement can be achieved by invoking the smoothing function

Figure 2 shows a typical plot of the counts $C_{\mathrm{ref},\lambda}$ versus $\lambda$. The pattern of this curve depends on the spectrum of the light source, the spectral sensitivity of the detecting elements (pixels) of the CCD, and the absorbance characteristics of the fibreoptic cables and of any light-intensity attenuator used. It is obvious that the spectral response is highly non-uniform, with best response expected in the spectral region of about 500–750 nm peaking at 621 nm. By doubling the integration time to 6 ms, pixels covering a wide spectral range will be saturated excluding any meaningful measurement of absorbance within this range. Quite a low response is observed at wavelengths lower than about 470 nm, resulting into a poor signal resolution which is responsible for the extremely high noise level observed across this spectral range (figure 1).

### *Intrinsic uncertainties of count measurements*

Random errors associated with count measurements are propagated and affect the precision of absorbance measurement, as it is obvious from equation (1). For the assessment of these uncertainties in terms of overall standard deviation, many measurements of counts were performed at various mean counts and wavelengths. In each case, a file of about 2500–3000 consecutive numbers of counts was generated and processed. The overall standard deviation increased as the mean number of counts increased, but not linearly. In all cases, a normal (Gaussian) distribution of this noise was observed. Typical frequency distributions of consecutive counts are shown in figure 3.

The noise components of a CCD are shot noise, dark signal and read-out noise. Shot noise was proportional to

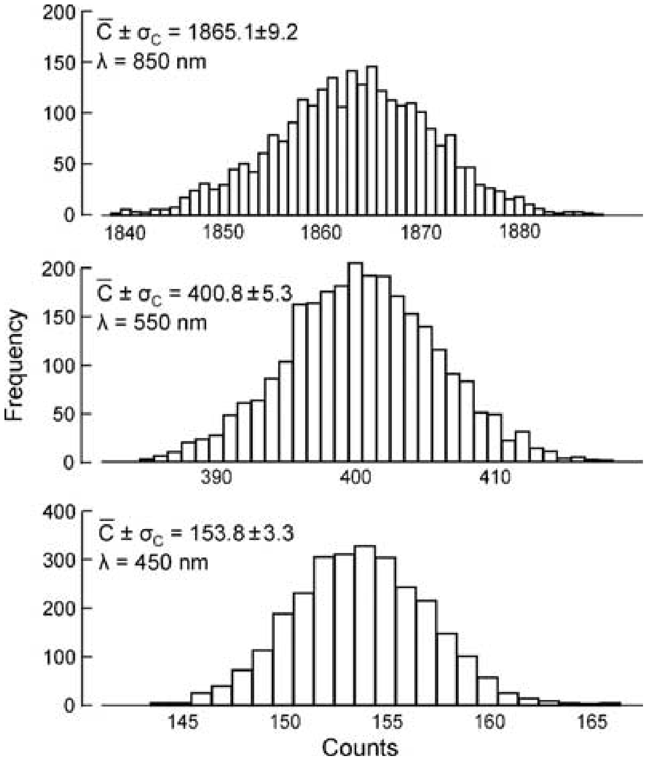

*Figure 3. Typical frequency distribution of a number registered* (n = *2740) of consecutive counts at three different mean count levels and three different wavelengths. Almost perfect Gaussian distributions are observed in all cases.*

the square root of the light signal, whereas only cooling can effectively reduce dark signal. Less than one electron per pixel and hour of a dark signal can be expected when the device is cooled with liquid nitrogen, making the CCD one of the most sensitive optical detectors [8]. Read-out noise is a function of the electronics and quality of the detector. A high number for the maximum count of the reference spectrum decreases the relative size of quantisation noise and expands the dynamic range of the absorbance measurements. Typically, an absolute maximum absorbance reading of $-\log(1/4095) = 3.6$ AU can be expected with a 12-bit A/D converter when the integer number of counts is used. Actually, by employing dithering and averaging techniques, this limit can be exceeded. Mechanical vibrations and light-intensity fluctuations contribute considerably to this random noise, but they are mostly sources of inaccuracy, as they may cause drift and unexpected signal stepwise changes particularly obliterating for measurements obtained with a single-channel device and based on prestored reference and dark spectral information.

Plots of the overall standard deviation of counts ($\sigma_C$) as a function of the counts number ($C$), obtained at four different wavelengths, are shown in figure 4. These plots reveal a non-linear dependence, which is described well by the following function:

$$\sigma_C = BC^m. \qquad (2)$$

Regression data obtained by a non-linear least-squares fit of equation (2) on the data shown in figure 4 are given in table 1.

*Table 1. Regression data of the non-linear least squares fit.*

| Wavelength (nm) | *B* | *m* | *r* |
|---|---|---|---|
| 450 | 0.4112 | 0.4041 | 0.996 |
| 550 | 0.4362 | 0.3931 | 0.98 |
| 650 | 0.3817 | 0.4069 | 0.98 |
| 750 | 0.4740 | 0.3984 | 0.99 |
| 850 | 0.4994 | 0.4115 | 0.994 |

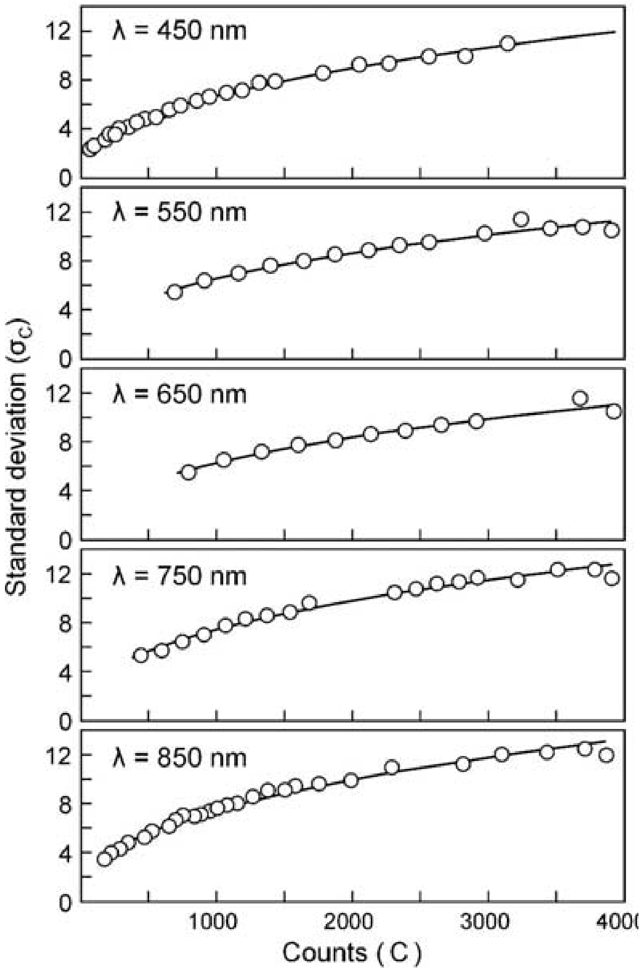

*Figure 4. Plots of the standard deviation of counts as a function of the counts at four different wavelengths.*

From the regression data shown in table 1, it seems that the exponent $m$ is practically constant with an average $0.4006 \pm 0.006$. This is close to the theoretically expected 0.5 for shot noise, indicating the prevalence of this noise in absorbance measurements. The pre-exponential term $B$ would normally be constant, but probably due to CCD minor structural asymmetries it was dependent loosely on the wavelength. This dependence can only roughly be approximated by the quadratic equation ($\lambda$ in nm) $B = 1.054 \times 10^{-6}\lambda^2 - 0.00116\lambda + 0.726$ ($r = 0.83$). Therefore, all regression data can be included in a single equation describing the expected standard deviation of counts number as a function of the actual counts level and the wavelength ($\lambda$ in nm)

$$\sigma_C(C, \lambda) = (1.054 \times 10^{-6}\lambda^2 - 0.00116\lambda + 0.726)C^{0.4006}. \quad (3)$$

The average absolute difference between the standard deviation calculated by equation (3) and the actual one was only about 0.7 counts over the spectral range 450–850 nm and the counts range 10–4000.

*Program for the prediction of absorbance precision patterns*

A computer program was developed for the calculation and drawing of contour plots showing the expected $RSD\%_A$ as a function of the mean absorbance and wavelength of the measurement. The basic input parameters are as follows.

- File containing the spectral response factors, i.e. the reference counts observed for each wavelength over the 350–850 nm range, as those described by figure 2. After the loading of this file, its values are normalized and a vector of spectral response factors ($a_\lambda$) is created, with $a = 1$ at the wavelength of maximum sensitivity (in the present case $\lambda_{max} = 621$ nm).
- Maximum number of counts ($C_{max}$) measured at $\lambda_{max}$ with the reference solution (typically within the 3800–3900 range, for a 12-bit analogue-to-digital converter, allowing a 200–300 counts safety margin to prevent saturation by overexposure).
- Equation describing the standard deviation of counts as a function of counts number and wavelength (equation 3).
- Average counts obtained during the dark measurement, $C_{dark}$. Since, $C_{dark}$ was found more or less constant over the entire wavelength range, it was entered as a single arithmetic parameter (typically within the range 40–60).

The expected standard deviation of absorbance as a function of absorbance and wavelength can be obtained by applying the propagation of random error mathematics on equations (1) and (3). Still, considering that raw measurements of counts are discrete (integers) and not continuous quantities, to make the program more realistic, flexible and ready to accept any type of equation describing the function $\sigma_C(C, \lambda)$, we applied a Monte Carlo simulation for the non-deterministic calculation of the expected absorbance uncertainties as a function of absorbance measurement and wavelength. More specifically, to calculate $RSD\%_A$ at any particular point of the A-$\lambda$ plane, the following steps took place.

(1) Expected reference count $C_{ref,\lambda}$ was calculated by the following equation:

$$C_{ref,\lambda} = a_\lambda C_{max} \quad (4)$$

where $a_\lambda$ is the spectral response factor at the specified wavelength ($0 \leq a_\lambda \leq 1$). $C_{ref,\lambda}$ is 'contaminated' with normal (Gaussian) noise and then it is rounded, as shown in the following equation:

$$(C_{ref,\lambda})_{noisy} = ROUND(NRANDOM(C_{ref,\lambda}, \sigma_C(C_{ref,\lambda}))), \quad (5)$$

where $NRANDOM$(MN, SD) is a function that generates normally distributed pseudo-random real numbers with mean MN and standard deviation SD based on a commonly used algorithm [9]. This algorithm uses uniformly distributed pseudo-random numbers (within the 0–1 range) generated by the standard function *RANDOM* found in all high-level computer languages and generates pseudo-random numbers normally distributed. Here, MN is $C_{ref,\lambda}$ and the SD is given by equation (3). To simulate more closely the actual system behaviour, all calculated counts were rounded by the standard function *ROUND*(X), which rounds the real number $X$ to the closer integer. It must be stressed that roundings from a statistical point of view correspond to 'contamination' with quantisation noise.

(2) Similarly, a dark count $(C_{dark,\lambda})_{noisy}$ 'contaminated' with normal noise is calculated using the following equation:

$$(C_{dark,\lambda})_{noisy} = ROUND(NRANDOM(C_{dark,\lambda}, \sigma_C(C_{dark,\lambda}))), \quad (6)$$

(3) Based on equation (1), the expected sample count $C_{sample,\lambda}$ for the given absorbance $A$ is calculated using the following equation:

$$C_{sample,\lambda} = (C_{ref,\lambda} - C_{dark,\lambda}) \times 10^{-A} + C_{dark,\lambda}, \quad (7)$$

where $C_{sample,\lambda}$ is similarly 'contaminated' with normal noise and rounded using the following equation:

$$(C_{sample,\lambda})_{noisy} = ROUND(NRANDOM(C_{sample,\lambda}, \sigma_C(C_{sample,\lambda}))), \quad (8)$$

(4) Absorbance 'contaminated' with noise is calculated from the equation:

$$A_{noisy}(A, \lambda) = -\log\left[\frac{(C_{sample,\lambda})_{noisy} - (C_{dark,\lambda})_{noisy}}{(C_{ref,\lambda})_{noisy} - (C_{dark,\lambda})_{noisy}}\right]. \quad (9)$$

Steps (3) and (4) are repeated $N_{sim}$ (number of simulations) times (typically $N_{sim} = 1000$) and an $RSD\%_A(A, \lambda)$ is calculated from all $N_{sim}$ $A_{noisy}(A, \lambda)$ values. The greater $N_{sim}$ is, a more precise $RSD\%_A(A, \lambda)$ is obtained. However, steps (1) and (2) are executed only once, since in actual consecutive measurements of absorbance, reference and dark data counts are similarly read and stored only once before the commencement of the absorbance measurements.

To obtain the contour plots describing the $RSD\%_A$ on the A-$\lambda$ plane, both the wavelength range 350–850 nm and absorbance range (0–2.5) were divided in 100 segments, resulting in an overall plot resolution of $100 \times 100$. Therefore, a typical Monte Carlo model (for $N_{sim} = 1000$) requires the calculation of $100 \times 100 \times 1000 = 10^7$ $A_{noisy}(A, \lambda)$ values, a computational task typically completed within 20–25 s on a Pentium 4 (1.7 GHz) computer.

Figure 5a shows the contour plot of the predicted $RSD\%_A$ by the Monte Carlo simulation, while figure 5b shows the plot generated by using actual experimental $RSD\%_A$. Figure 5b shows the plot generated by using

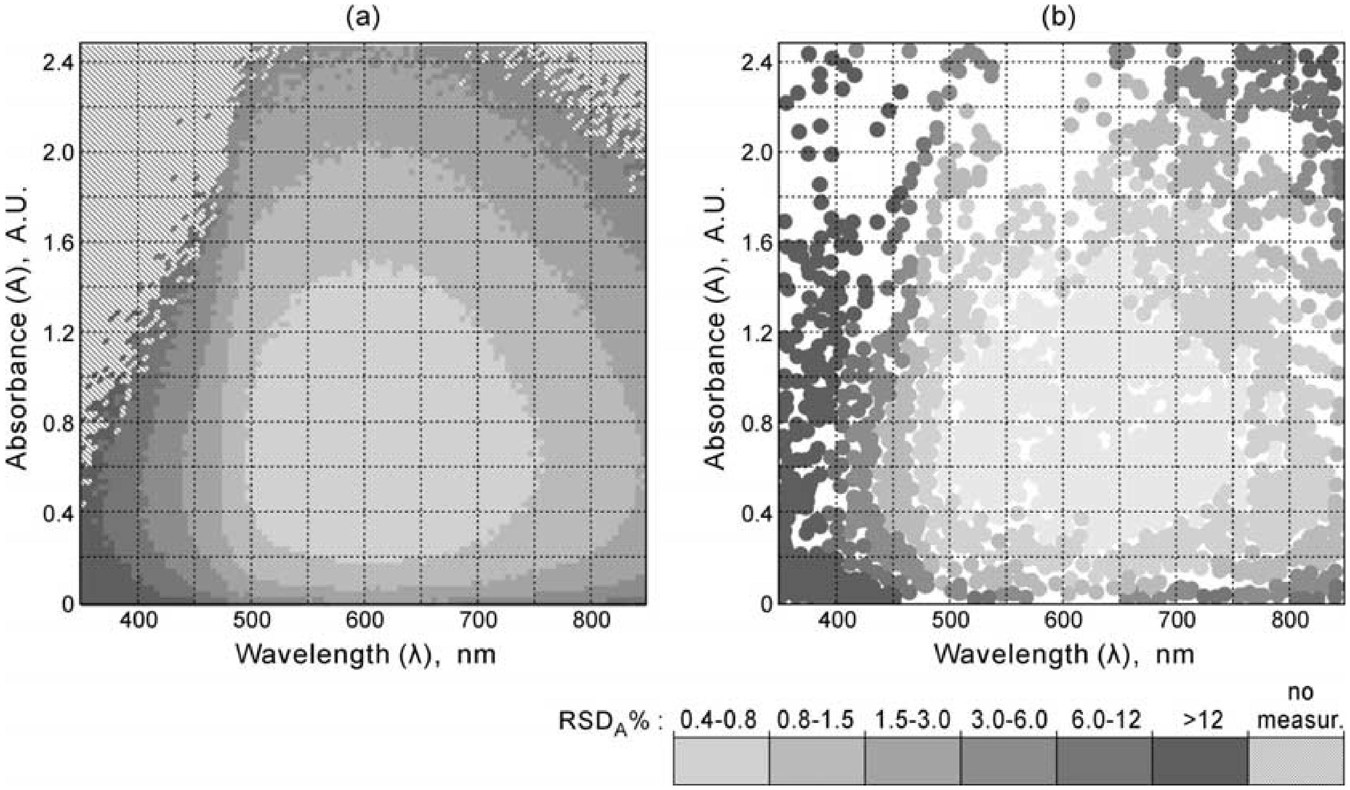

*Figure 5. Contour plots showing the expected relative standard deviation as a function of mean absorbance and wavelength. (a) Plot predicted by the Monte Carlo simulation model (input parameters: spectral response factors shown in the plot of figure 1,* $C_{max} = 3850$, $C_{dark,\lambda=} 50$, $N_{sim} = 1000$*); (b) plot created by using experimental data.*

actual experimental RSD%$_A$. The similarity of these plots supports the reliability of the Monte Carlo model. Both simulated and experimental data did not reveal RSD%$_A < 0.4$ under the aforementioned conditions of absorbance measurement.

The model seems less accurate at wavelengths below 400 nm where slightly more precise absorbance measurements are predicted than those experimentally obtained. Furthermore, in the unfavourable case of low spectral response combined with high absorbances, the difference $(C_{\text{sample}})_{\text{noisy}} - (C_{\text{dark}})_{\text{noisy}}$ may occasionally acquire a non-positive value making impossible the calculation of $A_{\text{noisy}}(A, \lambda)$. These 'forbidden' regions (mainly located at $\lambda < 500$ nm and $> 750$ nm) are properly marked in the contour plot of figure 5a. For example, the model predicts that consistent absorbance measurements higher than 1.4 at $\lambda = 400$ nm or smaller cannot be expected. Scattered measurements achieved in these regions are merely due to favourably low $(C_{\text{dark}})_{\text{noisy}}$ values randomly occurred at those particular A-$\lambda$ spots during the Monte Carlo simulation.

Despite that 'forbidden' regions on the A-$\lambda$ plane are predicted by the Monte Carlo model, the data-acquisition software (SpectraWin) keeps generating absorbances (even with high RSD%$_A$). Programming details of SpectraWin are not known, therefore we can only assume that this is achieved by some kind of error-trapping routine combined with a dithering approach that can generate absorbances.

*S/N enhancement: predicted effect of measurement parameters*

The CCD spectrometer tested offers several S/N enhancement options that can be applied alone or in combination. A typical enhancement option is based on averaging multiple scans. In this case, the RSD%$_A$ are statistically expected to be inversely proportional to $N_{\text{scans}}^{1/2}$, where $N_{\text{scans}}$ is the number of scans averaged. Figure 6 shows a typical plot of the obtained log(RSD%$_A$) versus log($N_{\text{scans}}$). A slope close to the theoretical 0.5 was obtained.

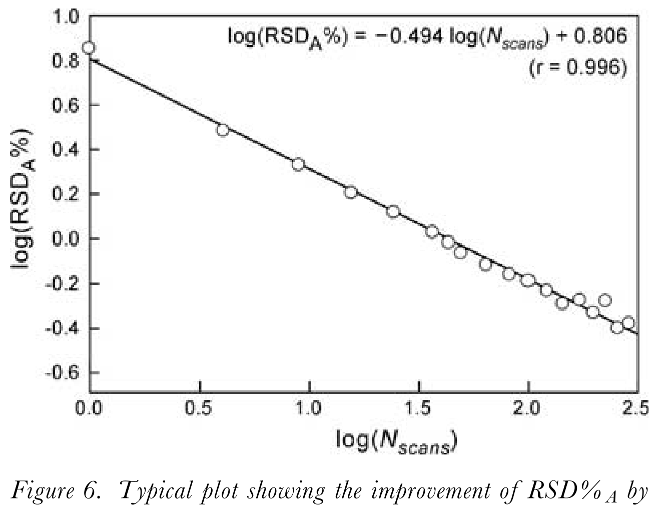

*Figure 6. Typical plot showing the improvement of RSD%$_A$ by averaging a number of scans (*$N_{\text{scans}}$*).*

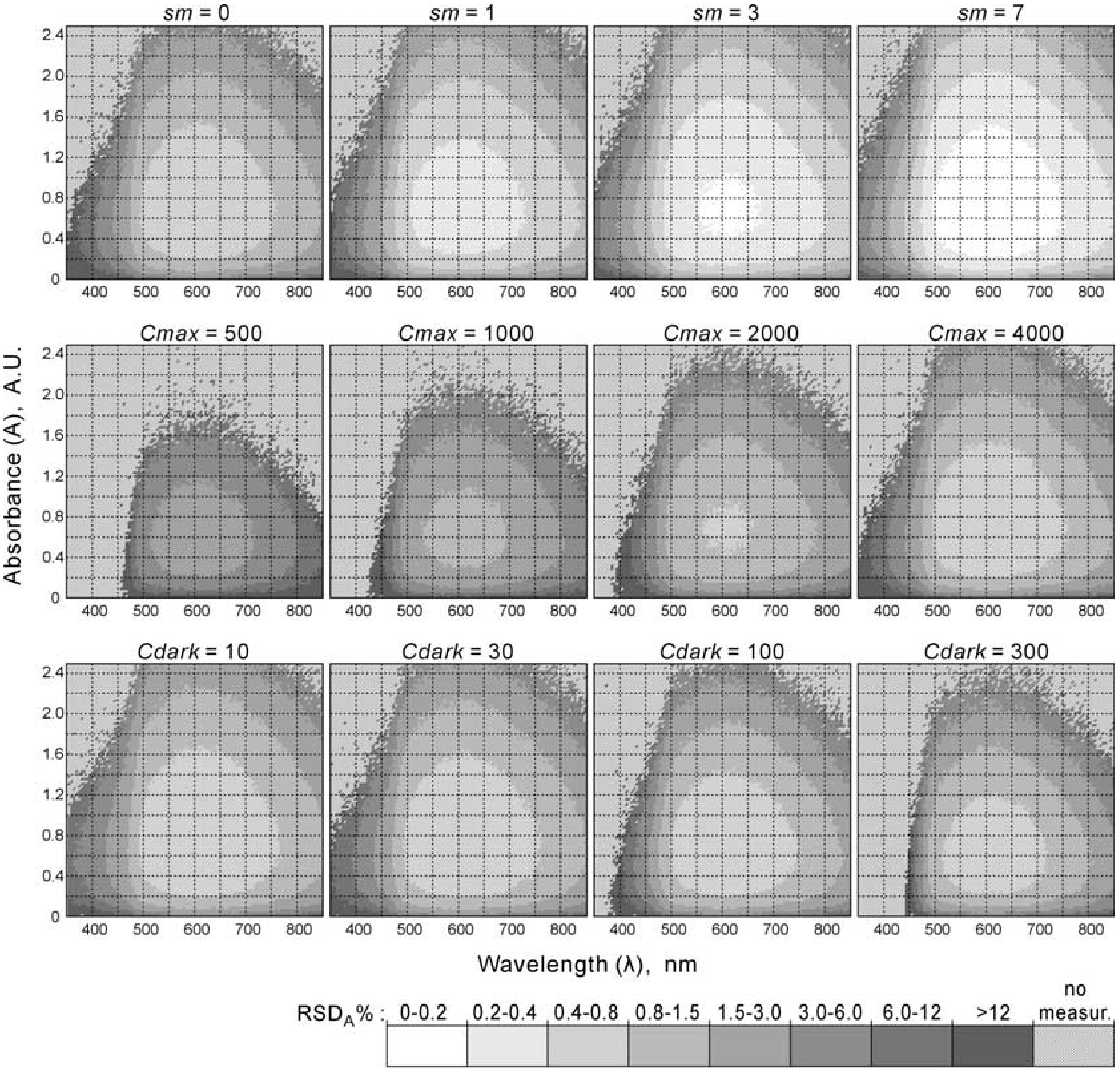

*Figure 7. Computer-generated contour plots showing the expected effect of various measurement parameters on the precision of the absorbance measurements. The standard parameters used (apart of the tested parameter shown above each plot) were:* sm = *0,* $C_{max}$ = *4000,* $C_{dark}$ = *50, no averaging.*

The main drawback associated with this signal enhancement approach is that averaging of too many scans makes the data acquisition a rather slow process. Unfortunately, the CCD detector cannot be instructed to perform scans over a limited number of adjacent pixels. All 2048 pixels are actually scanned and transferred serially to the computer, hence averaging of scans may be a relatively slow approach when fast data acquisition is needed, i.e. in the case of flow-injection analysers or in the case of sequential-injection systems performing determinations based on reaction rate measurements.

Another S/N enhancement feature is based on the increasing of integration time. This approach effectively reduces the shot noise component, which is inversely proportional to the square root of the observation period. Light integration is faster than averaging, because data are transferred only once to the computer so the time overhead is less than that required for scan averaging. A serious drawback is that spectral regions around the maximum detector sensitivity will be overexposed, therefore this approach cannot be applied when absorbances are measured at many wavelengths, and especially when whole spectra are required.

Probably, the most elegant feature of the data-acquisition program for S/N enhancement is data smoothing. It should be stressed that this is not an exclusively software-based smoothing approach of merely cosmetic value [10], but it is based on an increased influx of information. The pixels of the CCD are so closely packed that depending on the optical fibre diameter and/or entrance slit width, a number of adjacent pixels are practically illuminated by the same wavelength zone. Therefore, considering also that absorption peaks in the UV/Vis wavelength region are generally wide, the count numbers of neighbouring pixels can be averaged without a significant deterioration (in most cases) of the spectral resolution. By adjusting the smoothing parameter to a value *sm*, the number of counts of the central pixel is averaged with the *sm* pixels to the left and *sm* pixels to the right, hence $2sm+1$ counts are averaged, improving S/N by a factor of about $(2sm+1)^{1/2}$. This S/N enhancement feature has not any of the drawbacks described before.

The effect of certain parameters on the expected $RSD\%_A$ as it is predicted by the simulation program can be seen on the contour plots shown in figure 7.

In the upper row of contour plots shown in figure 7 is shown the effect of increasing the smoothing factor *sm*. For example, highly precise measurements ($RSD\%_A < 0.2$) can be obtained with $sm = 7$ over the wavelength range 500–750 nm and absorbance range 0.2–1.4. In the middle row is shown the expected adverse effect of poor adjustment of the $C_{max}$, whereas in the lower row the effect of increased $C_{dark}$ is displayed. $C_{dark}$ is not an adjustable parameter, but depends on the CCD quality and the actual operating temperature.

### Conclusions

The precision of absorbance measurements obtained by inexpensive single-channel spectrometers using a CCD detector depends strongly on the wavelength and absorbance. Fortunately, there are several ways of improving the precision that can be automatically invoked and applied only when needed by an appropriately designed data-acquisition program based on a prior knowledge of the expected precision. Thus, an almost constant precision of absorbance measurements can be obtained all over the absorbance-wavelength plane without compromising the data-acquisition rate.

### Acknowledgements

The authors are very grateful for the financial support of the Special Research Account of the National and Kapodistrian University of Athens, which made possible the purchase of the tested spectrometer.

### References

1. Rocha, F. R. P., Martelli, P. B. and Reis, B. F, *Anal. Chim. Acta*, **438** (2001), 11.
2. Wang, R. Y., Jarratt, J. A., Keay, P. J., Hawkes, J. J. and Coakley, W. T., *Talanta*, **52** (2000), 129.
3. Segura-Carretero, A., Rodriguez-Fernandez, J. Bowie, A. R. and Worsfold, P. J., *Analyst*, **125** (2000), 387.
4. Shultz, L. L., Stoyanoff, J. S. and Nieman, T. A., *Anal. Chem.*, **68** (1996), 349.
5. Ruzicka, J., *Analyst*, **125** (2000), 1053.
6. Coles, S., Nimmo, M. and Worsfold, P. J., *J. Automated Meth. Manag. Chem.*, **22** (2000), 97.
7. AVANTES, *Fiberoptic Spectroscopy Operating Manual*, Version 1.3, Eerbeek, The Netherlands (April 2001), 42.
8. Jobin Yvon/SPEX, *Guide for Spectroscopy* (Edison: Jobin Yvon/SPEX, 1994), 216.
9. Press, W. H., Flannery, B. P., Teukolsky, S. A. and Vettering, W. T., *Numerical Recipes: The Art of Scientific Computing* (Cambridge: Cambridge University Press, 1988), 202.
10. Enke, C. G. and Nieman, T. A., *Anal. Chem.*, **48** (1976), 705A.

